# Understanding Carbon Nanotube Voltammetry: Distinguishing Adsorptive and Thin Layer Effects via “Single-Entity” Electrochemistry

**DOI:** 10.1021/acs.jpclett.2c01500

**Published:** 2022-06-13

**Authors:** Archana Kaliyaraj Selva Kumar, Richard G Compton

**Affiliations:** Department of Chemistry, Physical and Theoretical Chemistry Laboratory, Oxford University, South Parks Road, Oxford, OX1 3QZ, Great Britain

## Abstract

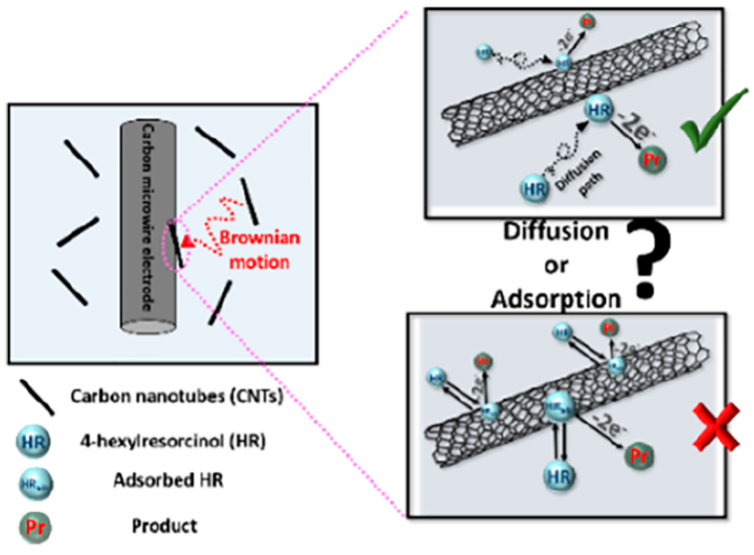

Cyclic voltammetry of ensembles of nanotube-modified electrodes fails to distinguish between signals from electroactive material adsorbed on the tubes from those due to a thin-layer response of analyte material occluded in the pores of the ensemble. We demonstrate that the distinction can be clearly made by combining cyclic voltammetry with single-entity measurements and provide proof of concept for the case of b-MWCNTs and the oxidation of 4-hexylresorcinol (HR), where the increased signals seen at the modified electrode are concluded to arise from thin-layer diffusion and not adsorptive effects. The physical insights are generic to porous, conductive composites.

Important and diverse roles are played by electrodes made of composites of carbon nanotubes (CNTs) or of surfaces modified, for example, by drop-casting layers of CNTs. Specifically, the areas of electroanalysis and energy conversion benefit from the use of these deliberately designed interfaces as can be judged by the selected examples given in Tables 1 and 2. From this, it can be inferred that CNT ensembles play a major role in electrocatalysis to benefit sensors, fuel cells, and batteries. Physically whether formed via drop-casting or some means of compression, perhaps with the addition of a binding agents such as Nafion,^1^ the electroactive layer usually takes the form of a random aggregate of CNTs and displays a significant level of porosity, although carefully grown nanotube “forests” confer greater order. Nevertheless, porosity is essential since it allows the access of electrolytes to the high internal surface area of the porous CNT layer which is at least partly the cause of the apparent electrocatalysis often shown by such surface modifications.^2^

**Table 1:** Examples of CNTs Modified Electrodes in Electroanalysis

CNTs modified electrode	application/technique used	refs.
multiwalled carbon nanotubes (MWCNTs) modified glassy carbon (GC) electrodes	detection of 2,4,6-trinitrotoluene via adsorptive stripping voltammetry.	(14)
GC modified with MWCNTs and ionic liquids	detection of ciprofibrate using differential pulse voltammetry	(15)
MWCNTs modified screen printed electrodes	detection of hesperidin using adsorptive stripping voltammetry.	(16)
MWCNTs modified GC electrode	detection of hydrocholothiazide (HCT) and triamterene (TRT) using stripping voltammetry.	(17)
Au nanoparticles deposited CNTs modified GC electrode	determination of As(III) using anodic stripping voltammetry	(18)
functionalized MWCNTs modified GC electrode	determination of paraquat using square-wave voltammetry	(19)

**Table 2:** Examples of CNTs Composite Electrodes in Batteries and Fuel Cells

CNTs electrode composite	application	ref
MWCNTs/tin oxide (SnO_2_) nanocomposite	anode material in microbial fuel cell	(21)
electrodeposited Pt–Ru and Pt–Ru–Ni nanoclusters on MWCNTs.	electrocatalyst for oxygen reduction reaction (ORR) and methanol oxidation reaction (MOR)	(22)
Pd deposited MWCNTs	anode material in direct formic acid fuel cell	(23)
graphene oxide/MWCNTs nanocomposite	electrocatalyst for vanadium redox flow battery	(24)
RuO_2_ modified CNTs	cathode in Li–O_2_ wearable battery	(25)
nitrogen doped CNTs	cathode material for lithium-air batteries	(26)
single-walled carbon nanotube (SWCNT) doped with nitrogen and phosphorus.	bifunctional oxygen electrocatalyst	(27)

Key to understanding the electrocatalytic responses of CNT modified electrodes is deciphering their voltammetry which is the current response to an electrode potential ramp applied to the CNT modified electrode. For the case of a flat, planar electrode this response is well understood and characterized by the Randles–Ševčík equations.^3−6^ Here, the peak current reflects a compromise between an increasing rate of electron transfer and an ever-decreasing concentration of reactant local to the electrode as the potential is scanned, and scales directly with the square root of scan rate, signaling the role of diffusion in bringing fresh reactant to the surface.^7^ In the case of electrodes modified with a porous layer, two peaks may be observed in the voltammetry. One peak again reflects semi-infinite diffusion to the exterior of the layer as for a flat nonporous surface, and the other seen at a lower overpotential results from the electrolysis of the reactant occluded within the porous layer which typically displays characteristics of “thin-layer” diffusion.^2,8−13^ The latter has a peak current which scales more directly with scan rate signifying that the contents of the porous layer are more responsive to alterations of potential and hence are relatively more under thermodynamic control, and the potential of the peak is closer to the formal potential of the redox couple so “catalyzed”.

It is evident that the response of the peak current to the scan rate can be used to signal the presence of thin-layer diffusion within a CNTs layer. However, if an analyte is adsorbed on the surface of a flat nonporous electrode the voltammetric signal also scales directly with scan rate,^20^ again reflecting the absence of diffusion. This then poses a question that if a voltammetric signal is observed from a porous layer of CNTs which scales directly with scan rate, does it signify that there is thin-layer diffusion within the porous layer or is the analyte adsorbed on the surface of the CNTs rather than being occluded within the pores?

The problem of differentiating between the diffusional and adsorptive behaviors of the CNT layers is not only of fundamental importance in understanding the electrode reaction mechanism but also relevant for analytical understanding since CNT modified electrodes are extensively used for adsorptive stripping analysis (see Table 1). In this application the CNTs are used to adsorptively pre–concentrate the analyte prior to being detected and quantified voltammetrically.^28−30^

In the following we compare and contrast ‘single-entity’ electrochemistry measurements with ensemble responses to make a clear distinction between adsorptive and thin-layer effects. Specifically, we consider the voltammetry of 4-hexylresorcinol at both the single bamboo-like multiwalled carbon nanotubes (b-MWCNTs) and at drop-casted layers of b-MWCNTs. The Fourier transform infrared (FTIR) studies of the b-MWCNTs obtained from the manufacturer^31^ are given in the Supporting Information (SI), section 2. The oxidation of HR is thought^30^ to be a two-electron and two-proton transfer process as given in Scheme 1.

**Scheme 1:**
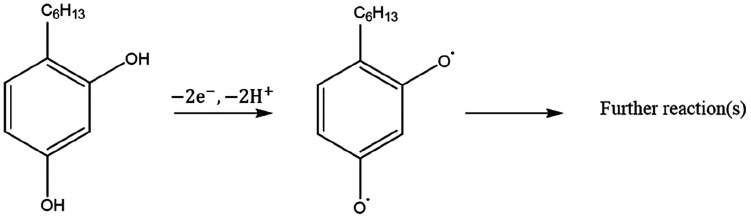
Electrochemical Oxidation of 4-Hexylresorcinol Undergoing a Two-Electron and Two-Proton Process^30^

The first voltammetric investigations were conducted using 0.1 mM aqueous solutions of 4-hexylresorcinol (HR) with 0.05 M Britton-Robinson buffer solution (BRS) of pH 1.5. The cyclic voltammograms (CV) were recorded as a function of scan rate at a pristine glassy carbon (GC) electrode, and at drop-casted ensembles (preparation of electrodes given in SI, section 1) of b-MWCNTs of different coverages (70, 98, 140, and 170 μg cm^–2^ of b-MWCNTs on GC). The voltammograms are given in SI, section 3, Figure S2. The number of layers of b-MWCNTs were estimated (SI, section 4), assuming that the nanotubes were arranged in a closed packing arrangement at the GC electrode surface, and were found to be 21, 30, 42, and 50 layers of b-MWCNTs for a drop-cast of 70, 98, 140, and 170 μg cm^–2^, respectively. Given the assumption made with respect to the packing, these numbers are likely to be significant underestimates since the layers will in reality likely be made of randomly oriented tubes. The overlaid voltammograms at bare GC and different coverages of b-MWCNTs drop-casted GC at a scan rate of 100 mV s^–1^ are shown in Figure 1, where a chemically irreversible oxidative peak was observed at *ca.* + 0.74 V vs SCE, which matches well with the reported formal potential for oxidation of HR.^30^ Moreover, in the absence of HR, no such voltammetric feature was observed as shown in SI, section 5, Figures S3 and S4. The plots of peak current versus scan rate and square root of scan rate are given in SI, section 6, Figure S5. To assess the scan rate dependence of the peak current, log–log plots were made as shown in Figure 2. Approximately linear plots were observed, the slopes of which are given in Figure 2, where slope values between 0.5 and 1.0 were noted. A slope of 0.5 would correspond to a semi-infinite diffusional signal while a value of 1.0 corresponds to adsorptive or thin layer responses. It can be seen that as the coverage of b-MWCNTs increases the signals tends toward unity indicating a greater contribution from the porous b-MWCNT layer as would be expected. It is evident that while the CV measurements emphasize the role of the porous layer they cannot in themselves distinguish between adsorptive and thin-layer effects. Accordingly, we next turn to ‘single-entity’ electrochemistry measurements.

**Figure 1:**
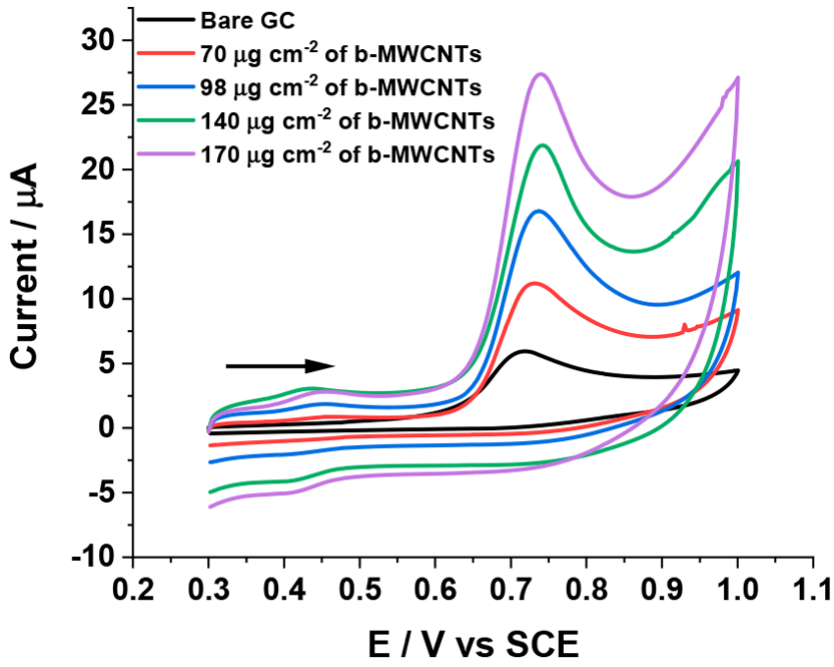
Cyclic voltammograms of 0.1 mM hexylresorcinol in 0.05 M BR buffer solution at bare GC electrode (black line), at variable drop-casted coverages of b-MWCNTs: 70 (red line), 98 (blue line), 140 (green line), and 170 (magenta line) μg cm^–2^ on a GC electrode (radius = 1.5 mm) at a scan rate of 100 mV s^–1^.

**Figure 2:**
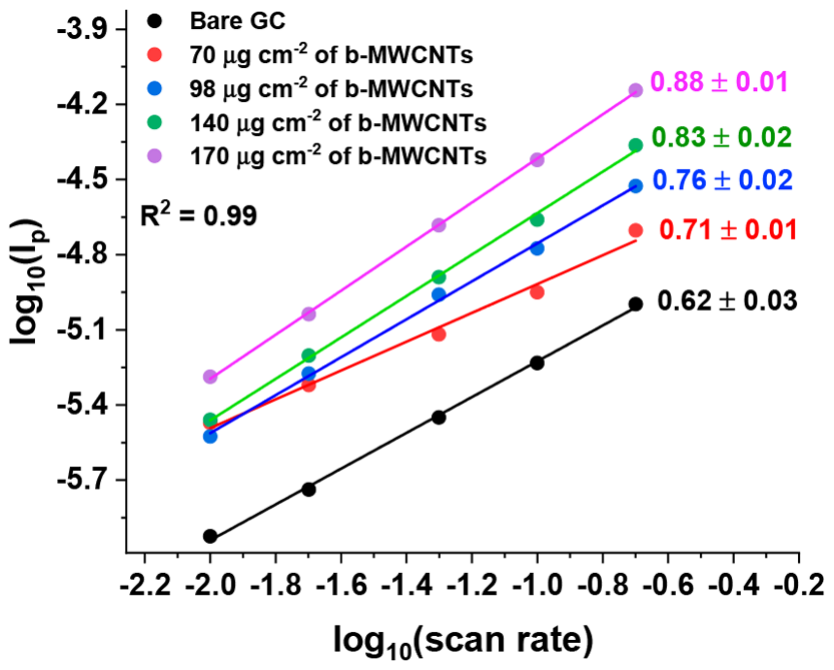
Plot of log peak current versus log scan rate in 0.1 mM hexylresorcinol with 0.05 M BR buffer solution at bare GC electrode (black circles), 70 (red circles), 98 (blue circles), 140 (green circles), and 170 (magenta circles) μg cm^–2^ of b-MWCNTs on a GC electrode.

Single-entity electrochemical analysis (also known as ‘nano-impacts’) of b-MWCNTs in 0.1 mM HR with 0.05 M BRS was carried out to understand the voltammetry at the single b-MWCNT levels without the complication of the uncertain mass-transport characteristics arising from layer porosity in the CV of drop-cast experiments as mentioned above. For this, chronoamperometric experiments were carried out at different fixed potentials using a carbon fiber microwire electrode (7 μm in diameter, for fabrication method see SI, section 1). The selected potential was applied to the electrode with 1.29 × 10^–14^ M of b-MWCNTs dispersed in it. Figure 3 shows representative impacts measured in the presence of 0.1 mM HR. The sharp on–off features correspond to the arrival and departure of the b-MWCNT from the surface of the electrode. Impact signals were measured over the potential range of +0.5 V to +1.0 V vs SCE, and the representative impacts are shown in SI, section 7, Figure S6. During the period of the impact, sustained Faradaic currents flow which are of an approximately constant value. Also some random current fluctuations are evident, which arise from movement of the b-MWCNT at the interface, modulating the electron transfer distance.^32^

**Figure 3:**
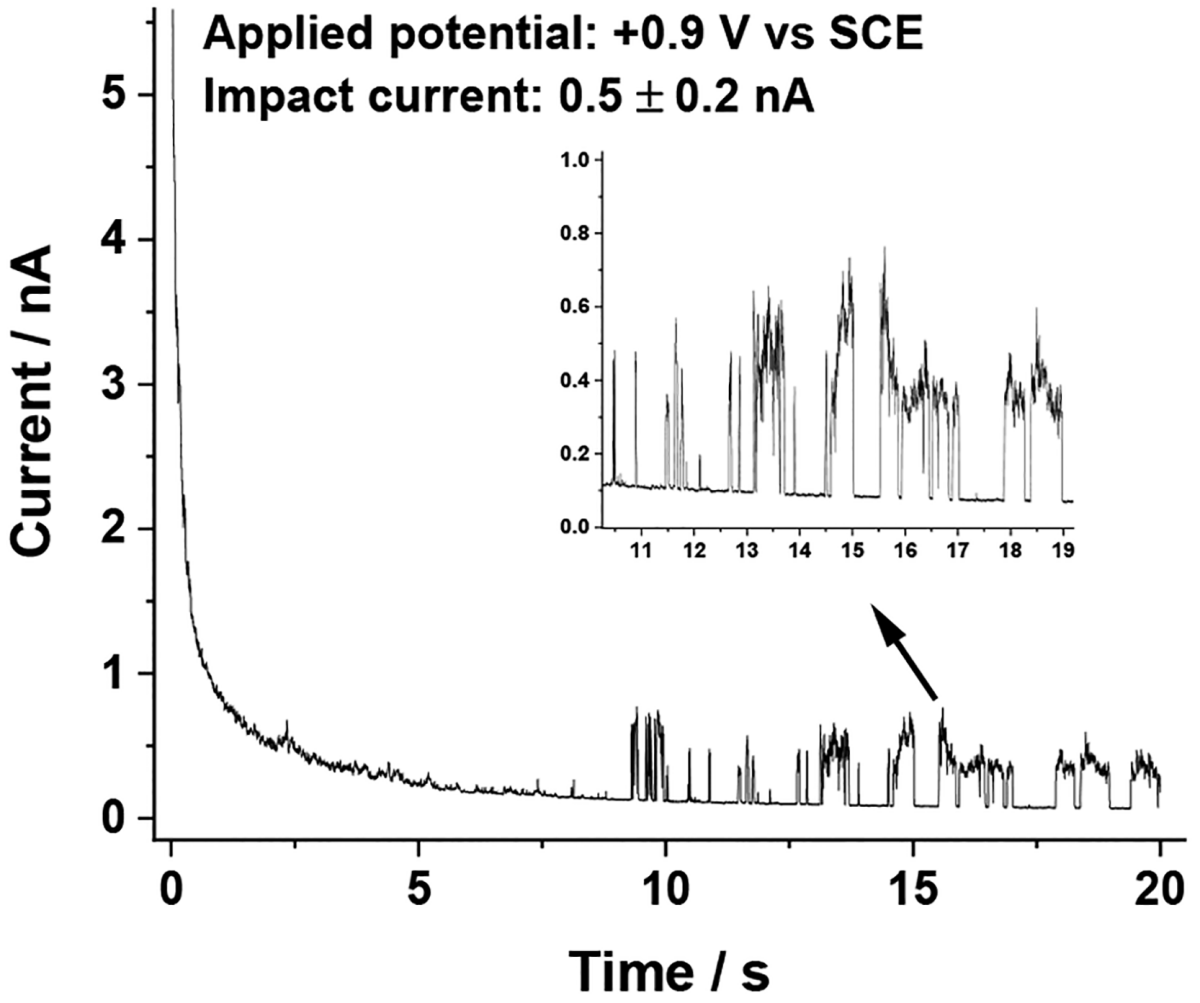
Chronoamperogram of 0.1 mM HR in 0.05 M BRS at an applied potential of +0.9 V vs SCE showing impact currents of 0.5 (±0.2) nA.

The red line in Figure 4a shows how the average impact current varies with potential leading to a voltammogram-like dependence with a maximum current seen as a plateau at potential above +0.9 V vs SCE. Comparison with Figure 1 suggests that the current likely arises from the oxidation of HR. The average impact duration was 1.2 s and the frequency of impacts 0.5 s^–1^. Note that no impact signals were seen in the absence of b-MWCNTs as shown in SI, section 8, Figure S7. In the absence of HR, but in the presence of the b-MWCNTs, tiny signals were observed, and the average impact current is shown by the black circles in Figure 4a. Figure S8 shows typical impact signals seen in the absence of HR at +0.9 V to +1.4 V vs SCE. These were assigned to capacitive impacts consistent with the increase in capacitive currents at high positive potentials^33,34^ and also the oxidation of the b-MWCNTs themselves as discussed elsewhere.^35^ The impact signals seen in the absence of HR are tiny in comparison with the Faradaic signals seen in the presence of HR; hence, attention was next turned to the further consideration of these signals.

**Figure 4:**
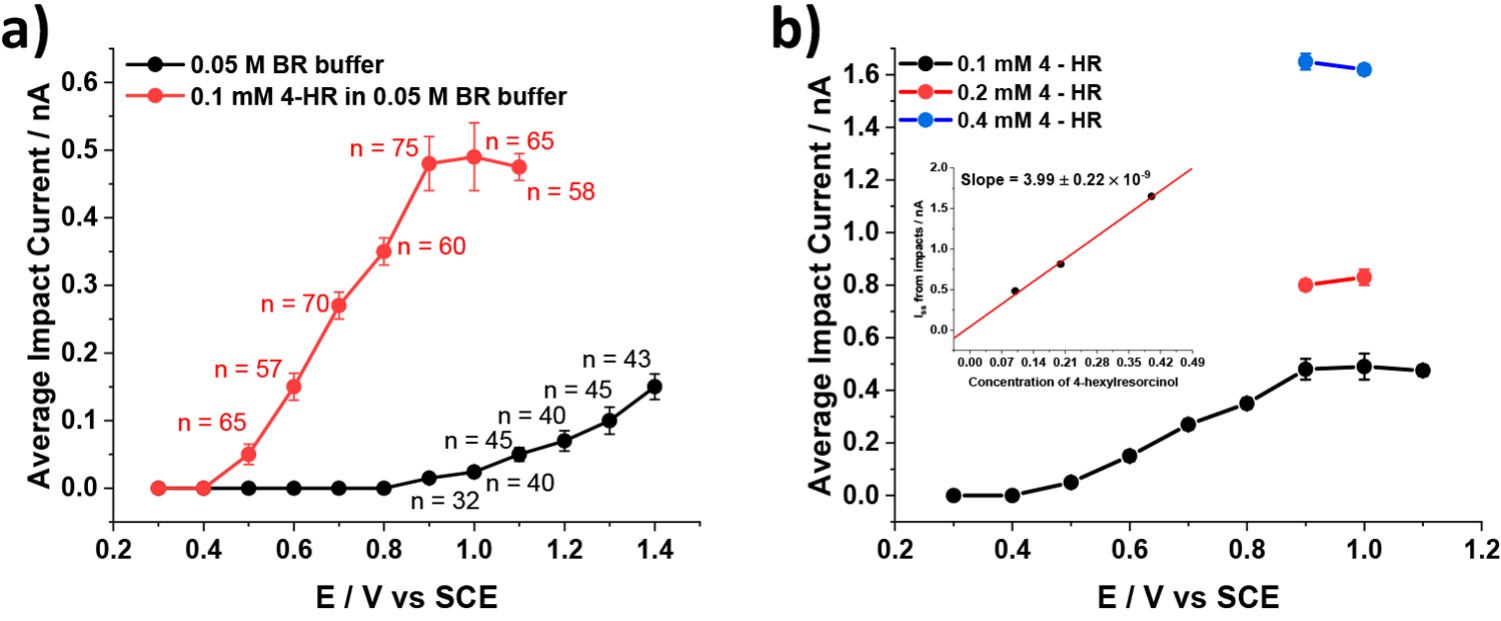
Plot of average impact current versus applied potential in b-MWCNTs dispersed (a) in 0.1 mM HR in 0.05 M BR buffer solution (red circles) and 0.05 M BR buffer solution only (black circles) where *n* is the number of impacts analyzed at each applied potential; (b) in 0.1 mM (black circles), 0.2 mM (red circles), and 0.4 mM (blue circles) of HR, in 0.05 M BRS.

Further experiments were conducted in which impact currents were measured as a function of potential using increased concentrations of HR of 0.2 and 0.4 mM, using potentials corresponding with the plateau current seen in Figure 4a for 0.1 mM HR. The data are shown in Figure 4b (red and blue circles for 0.2 and 0.4 mM, respectively) showing that the current scales approximately linearly with concentration. This observation is consistent with two possible explanations: first the signals represent the oxidation of HR adsorbed on the b-MWCNT surface or, second, the signals represent a diffusion-controlled oxidation of HR at the single b-MWCNT entity. The sustained currents flowing throughout the duration of the impact suggest the latter. Further in the first case the average charge per impact was calculated for each of the three concentrations of HR studied and compared with that expected for the 2-electron oxidation on the basis of an estimated monolayer coverage of HR on a single CNT. The estimation of the latter and the comparison is shown in the SI, section 10. It was found that if adsorption takes place then it must be multilayer adsorption on the single b-MWCNT as shown in the SI, Table S1 with as many as 4–20 layers observed depending on the orientation of HR at the b-MWCNT surface; this is probably unlikely.

Alternatively, the impact data was analyzed on the assumption that the steady currents reflect diffusion-controlled oxidation of HR. The magnitude of this can be estimated using an established model treating the single b-MWCNT as a cylindrical electrode^32,36−39^ using the known length and radius of the tube and assuming the oxidation is a 2-electron transfer process as in Scheme 1. This analysis is consistent with a diffusion coefficient for HR of 4.0 (±2.3) × 10^–6^ cm^2^ s^–1^ (calculations in SI, section 11).^40^ This value is of a magnitude expected for quinones in aqueous solutions (SI, Table S2). We infer that HR undergoes diffusional electro-oxidation at the b-MWCNTs when studied as single entities and hence infer that in the case of the CV of ensembles b-MWCNTs that the response reflects thin-layer diffusion, rather than adsorptive effects. Note that the multiwall nature of the CNTs does not have an effect on the single entity results since the interpretation is based on the solute diffusion to the exterior of the CNTs and is specific to the b-MWCNT studied in this work. Albeit, the effects of intercalation could be observed in others. Also, often phenolic and quinone groups are found to adsorb on the carbon surfaces including CNTs.^41−43^ In case of HR we speculate that the absence of adsorption is due to the bulky hexyl group attached to the resorcinol moiety.

To conclude, the oxidation of HR was investigated at both ensemble and “single” b-MWCNTs through voltammetric and “nano-impacts” methods, respectively. Voltammetric analysis of ensembles of b-MWCNTs were inconclusive in distinguishing between the adsorptive and thin layer diffusive effects. However, single entity measurements clearly demonstrated diffusion-controlled electrochemistry at individual carbon nanotubes, allowing the inference that the ensemble voltammetric response is that of thin layer diffusion from within the pores between the b-MWCNTs rather than resulting from adsorption onto the nanotubes. The schematic representation showing the experimental procedure is shown in Scheme 2, which summarizes the work. Last, we note that while this work was conducted on the b-MWCNTs the implications are generic to porous electrodes in general including the many used in energy conversion and chemical sensing based on nanoparticles.

**Scheme 2:**
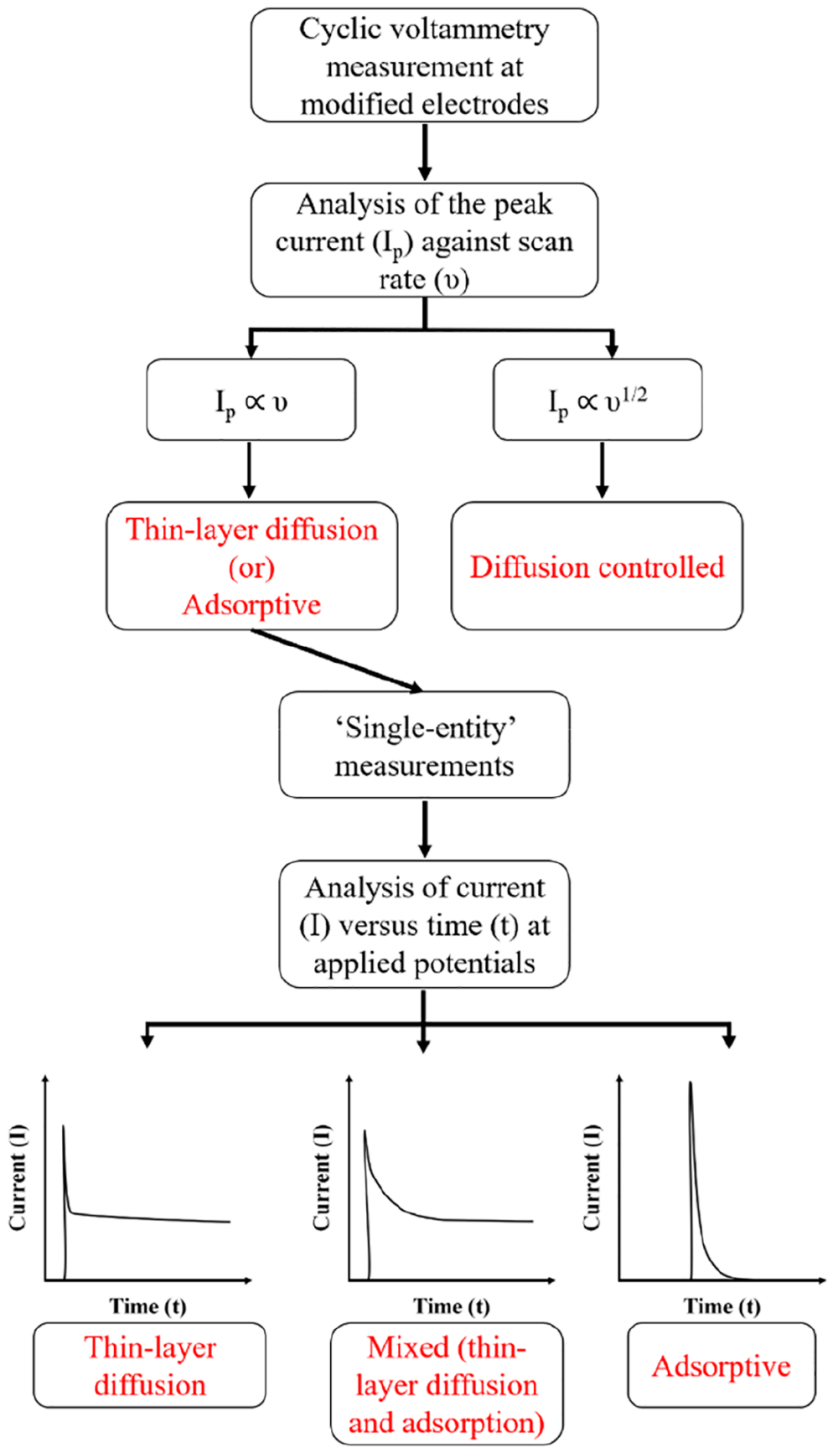
Schematic Representation of Experimental Procedure to Distinguish between Thin-Layer Diffusion and Adsorptive Voltammetry

## Supporting Information Available

Materials and methods, b-MWCNTs characterization, scan rate study at bare GC and b-MWCNTs drop-casted electrodes, estimation of monolayers of b-MWCNTs, cyclic voltammetry in the absence of 4-hexylresorcinol at bare GC and b-MWCNTs modified GC electrode, peak current dependence on scan rate and square root of scan rate study, chronoamperograms in 0.1 mM HR in 0.05 M BR buffer solution, in the absence of 4-hexylresorcinol, in 0.05 M BR buffer solution with b-MWCNTs, interpreting impact signals as adsorptive, interpreting impacts as diffusional signals from 4-hexylresorcinol, literature on the diffusion coefficients of quinones in aqueous solutions (PDF)
