# Supplementary material for: Understanding Carbon Nanotube Voltammetry: Distinguishing Adsorptive and Thin Layer Effects via “Single-Entity” Electrochemistry
Source: J Phys Chem Lett. 2022 Jun 13;13(24):5557–62. doi: 10.1021/acs.jpclett.2c01500 (PMC9234977; doi:10.1021/acs.jpclett.2c01500)
Supplement: Supplementary file 1 — jz2c01500_si_001.pdf [file jz2c01500_si_001.pdf]

Supporting Information

Understanding carbon nanotube voltammetry: Distinguishing adsorptive and thin layer effects via 'single-entity' electrochemistry

Archana Kaliyaraj Selva Kumar, Richard G Compton*

Department of Chemistry, Physical and Theoretical Chemistry Laboratory, Oxford University, South Parks Road, Oxford OX1 3QZ, Great Britain

* Corresponding author.
E-mail: richard.compton@chem.ox.ac.uk (Richard Compton)

Supporting Information

1. Materials and Methods
2. Characterisation of b-MWCNTs
3. Scan rate study at bare GC and b-MWCNTs drop-casted electrodes
4. Estimation of number of monolayers of b-MWCNTs on GC electrode
5. Cyclic voltammetry in the absence of 4-hexylresorcinol at bare GC and b-MWCNTs modified GC electrode
6. Peak current dependence on scan rate and square root of scan rate study
7. Chronoamperograms showing impact currents at various applied potential in 0.1 mM HR in 0.05 M BR buffer solution
8. 'Nano-impacts' in the absence of 4-hexylresorcinol
9. Chronoamperograms showing impact currents at various applied potential in 0.05 M BR buffer solution with b-MWCNTs
10. Interpreting impact signals as adsorptive in origin.
11. Interpreting impacts as diffusional signals from 4-hexylresorcinol
12. Literature on the diffusion coefficients of quinones in aqueous solutions

References

Section 1. Materials and methods

1.1 Materials

The chemicals used in this study were purchased commercially and used without any further purification. 4-hexylresorcinol, boric acid (H_3BO_3), phosphoric acid (H_3PO_4), acetic acid (CH_3COOH), and acetone >= 99.50 % were purchased from Merck Life Science, UK. Bamboo – like multiwalled carbon nanotubes (b-MWCNTs; diameter 30 (± 10) nm, length 5 - 20 μm and specific surface area of 200 m^2g^{-1}) were purchased from NanoLab, Inc., Massachusetts, USA. The b-MWCNTs consisted atomic % of 99.75 % of carbon, 0.05 % of sulphur and 0.20 % of iron. Solutions were prepared with deionised water (resistivity of 18.20 $M\Omega \cdot cm^2$, 298.15 K, Milllipore).

1.2 Cyclic voltammetry experiments

All the cyclic voltammetry (CV) were carried out in a Faraday cage at 298.15 K. The studies were conducted using a three-electrode cell comprising of a working electrode, a reference electrode (saturated calomel, SCE) and a counter electrode (graphite rod) in a μAutolab II potentiostat (Metrohm-Autolab BV, Netherlands). The voltammetry studies were controlled by General Purpose Electrochemical System (GPES) software. Bare glassy carbon (GC) and different coverages of b-MWCNTs (70, 98, 140 and 170 μg cm^{-2}) drop-casted on GC electrode were used as working electrodes for the cyclic voltammetry experiments.

1.2.1 Bare GC working electrode

A bare GC electrode of radius 0.15 cm and area 7.07 x 10^{-2} cm^2 was used as the working electrode. The GC electrode was polished sequentially with 1.00 μm, 0.30 μm and 0.05 μm alumina powder (Buehler, Micropolish II, Germany). Electrodes were then washed with deionised water and sonicated for 10 s then dried with N_2 prior to the CV experiments.

1.2.2 b-MWCNTs modified GC working electrode

A dispersion of b-MWCNTs was made in acetone 2.2 x 10^{-13} M (1 mg mL^{-1}), and sonicated for 20 mins. and dried under steady N_2 flow. 5, 7, 10 and 12 μg (70, 98, 140, 170 μg cm^{-2} in coverage respectively) of the above dispersion were drop-casted on the cleaned GC electrode and then dried under stable N_2 flow prior to the CV experiments.

1.5 'Nano-impacts' experiments on single b-MWCNT

The 'nano-impact' experiments were made at fixed potentials applied to the working electrode. A carbon micro-wire electrode of diameter 7 μm and length 1 mm (Goodfellow, Cambridge Ltd., UK) was employed as the latter. Fabrication of the micro-wire electrode was as reported in the literature [1]. The b-MWCNTs were dispersed in deionised water 2.2 x 10^{-13} M (1 mg mL^{-1}) and sonicated for 20 mins, then a known concentration was added to the solution of study for observing the impact siganls.

Section 2. Characterisation of b-MWCNTs

The Fourier Transform Infrared (FTIR) studies of b-MWCNTs obtained from the manufacturer is given in Figure S1. The peak at 3450 cm^{-1} is assigned to the -OH stretching in carboxyl groups (-COOH and =COH) and the peaks at 1615 cm^{-1}, and 1575 cm^{-1}, is assigned to C-C vibrations and C-O strectching vibrations due to internal defects of CNTs. The peak at 1575 cm^{-1} correspond to the characteristics backbone C=C skeletal stretching.

Transmission electron microscopy (TEM) showing 9 concentric walls and an empty, clear inner channel can be found in [2].

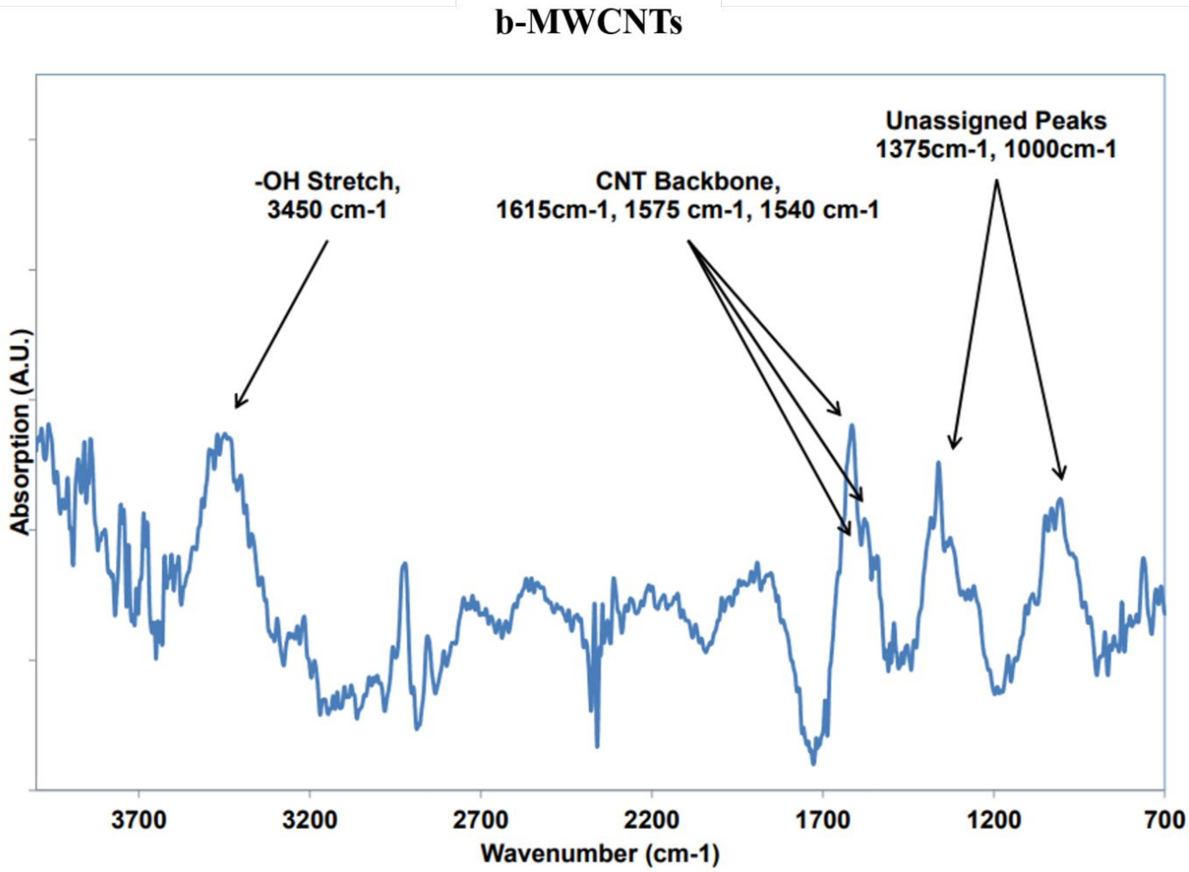

Figure S1. FTIR of b-MWCNTs from the manufacturer [2].

Section 3. Scan rate study at bare GC and b-MWCNTs drop-casted electrodes

Here, the cyclic voltammograms recorded as a function of scan rate in 0.1 mM 4-hexylresorcinol (HR) in 0.05 M Britton-Robinson buffer solution (BRS) of pH 1.5 at bare GC and different coverages of b-MWCNTs drop-casted on GC electrode is given.

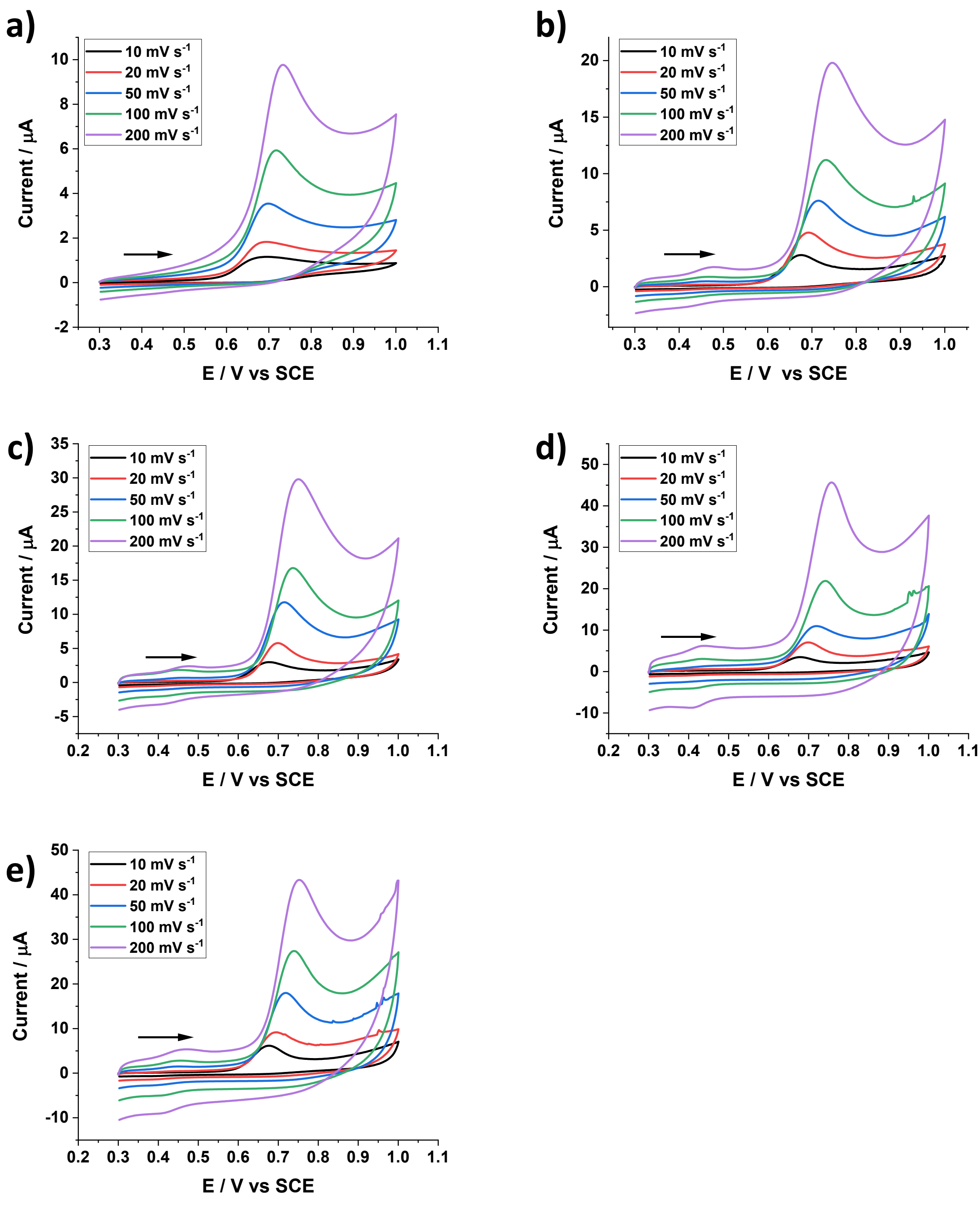

Figure S2. Cyclic voltammograms of 0.1 mM hexylresorcinol in 0.05 M BR buffer solution as a function of scan rate at a) bare GC, b) 70 µg cm^{-2} of b-MWCNTs on GC, c) 98 µg cm^{-2} of b-MWCNTs on GC, d) 140 µg cm^{-2} of b-MWCNTs on GC and e) 170 µg cm^{-2} of b-MWCNTs on GC.

Section 4. Estimation of number of monolayers of b-MWCNTs on GC electrode

To estimate the number of monolayers at the glassy carbon (GC) electrode for the drop-cast experiments we assume a closed pack arrangement of b-MWCNTs laid uniformly across the GC surface (area of GC electrode is 7.07 x 10^{-2} cm^2). The diameter of the b-MWCNTs is 30 nm, length is 6 – 20 μm and density is 1.4 g cm^{-3} (these values were sourced from the manufacturer).

Volume of single MWCNT (cylindrical geometry);

$$Volume = \pi r^2 l$$

where *r* is the radius of the b-MWCNT and *l* is the length of the b-MWCNT. Hence volume of single b-MWCNT is calculated to be 4.24 x 10^{-15} cm^3.

Then, mass of single b-MWCNTs

$$Mass = \ Density \ \times Volume \ of \ single \ b - MWCNT$$

Thus, the mass of single b-MWCNT is 5.94 x 10^{-15} g.

1 mg of b-MWCNTs was dispersed in 1 mL of acetone for the drop-cast experiments, hence 5 μL of drop-cast will contain 5.00 x 10^{-6} g of b-MWCNTs. Therefore, number of b-MWCNTs present in 5 μL drop-cast is found to be 8.33 x 10^8.

Assuming the contact at the GC surface by b-MWCNTs to be rectangular, when arranged in a closed pack manner, the area covered by single b-MWCNT is 1.80 x 10^{-9} cm^2. Hence, area covered by 8.33 x 10^8 b-MWCNTs is 1.50 cm^2. Now, that we know the total area of both GC electrode and that of b-MWCNTs hence, number of layers can be calculated as follows;

$$No. \ of \ monolayers \ of \ b - MWCNTs \ for \ 5 \ \mu L \ drop - cast = \frac{Total \ area \ covered \ by \ b - MWCNTs}{Total \ surface \ area \ of \ GC \ electrode}$$

Thus, number of layers for 5 μL drop cast on GC electrode is found to be 21 ± 2 monolayers.

Similarly, for a drop-cast of 7, 10 and 12 μL the numbers of monolayers were estimated to be 30, 42 and 50 layers of b-MWCNTs on GC electrode. As discussed in the main text these are likely to be significant under-estimates.

Section 5. Cyclic voltammetry in the absence of 4-hexylresorcinol at bare GC and b-MWCNTs modified GC electrode

In this section, cyclic voltammograms observed in the presence (0.1 mM HR in 0.05 M BRS) and absence (only 0.05 M BRS) of HR are compared and is shown at a scan rate of 10 mV s^{-1} and 100 mV s^{-1}.

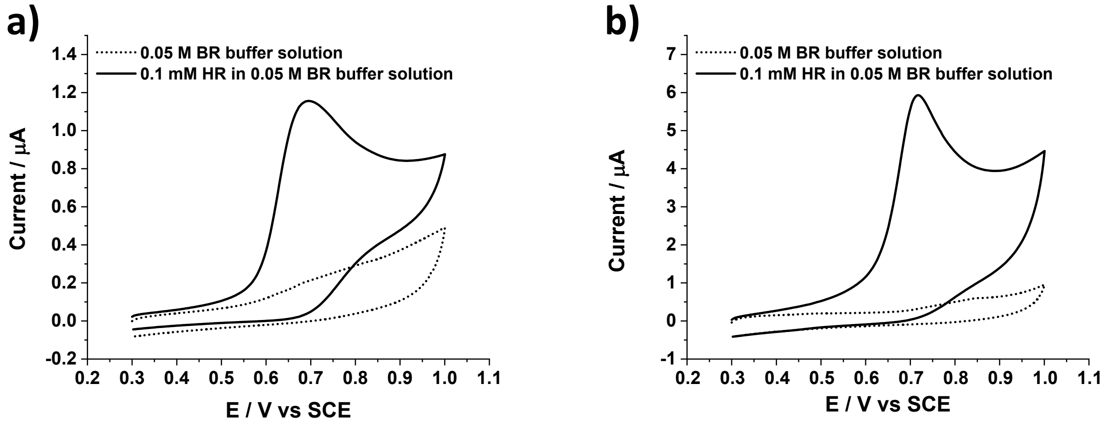

Figure S3. Cyclic voltammograms at bare GC electrode in 0.1 mM HR with 0.05 M BR buffer (solid line) and only BR buffer solution (dotted line) at scan rates a) 10 mV s^{-1} and b) 100 mV s^{-1}.

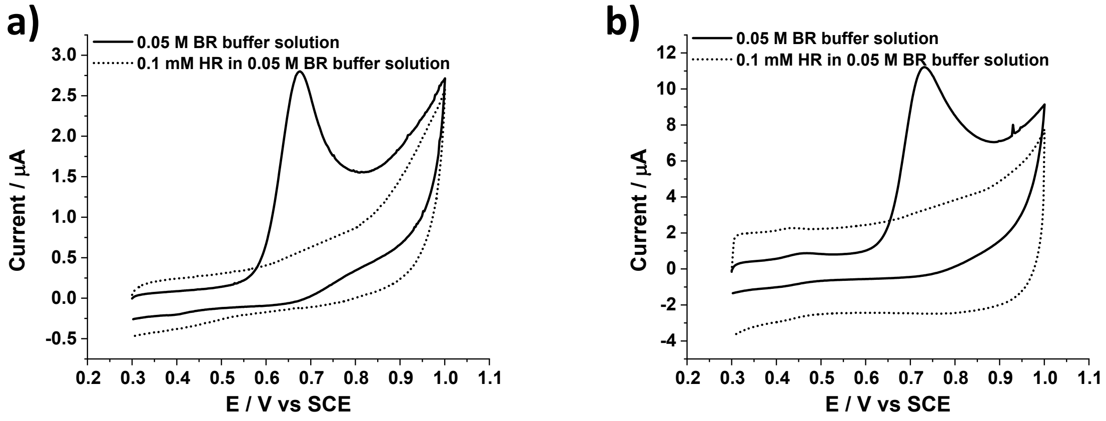

Figure S4. Cyclic voltammograms at 70 μg cm^{-2} of b-MWCNTs on GC electrode in 0.1 mM HR with 0.05 M BR buffer (solid line) and only BR buffer solution (dotted line) at scan rates a) 10 mV s^{-1} and b) 100 mV s^{-1}.

Section 6. Peak current dependence on scan rate and square root of scan rate study

In this section, the plot of peak current versus scan rate and square root of scan rate is given at bare GC electrodes and at different coverages of b-MWCNTs modified GC electrode.

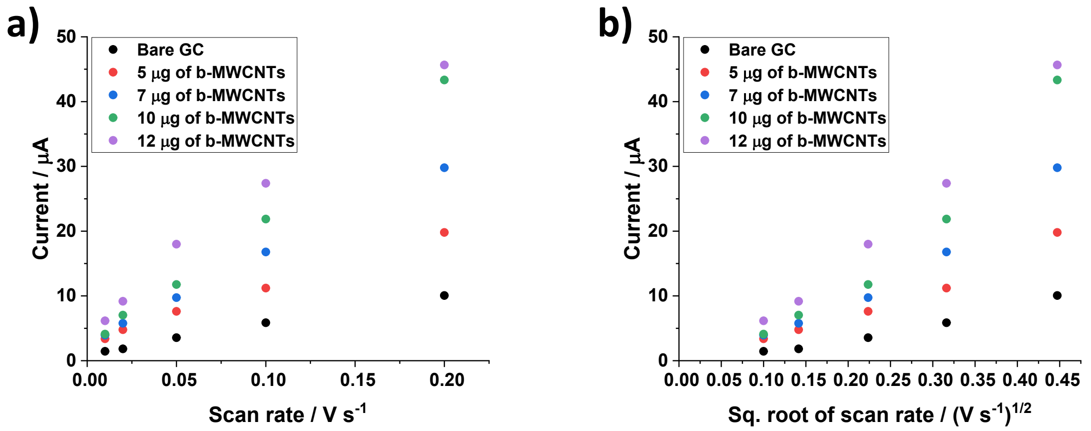

Figure S5. Bare GC electrode (black circles), 70 (red circles), 98 (blue circles), 140 (green circles) and 170 (magenta circles) μg cm^{-2} of b-MWCNTs on a GC electrode, in 0.1 mM hexylresorcinol with 0.05 M BR buffer solution. a) plot of peak current versus scan rate, b) plot of peak current versus square root of scan rate.

Section 7. Chronoamperograms showing impact currents at various applied potential in 0.1 mM HR with 0.05 M BR buffer solution

In this section illustrative chronoamperograms showing impact signals in 0.1 mM HR with 0.05 M BR buffer solution is given at different applied potentials.

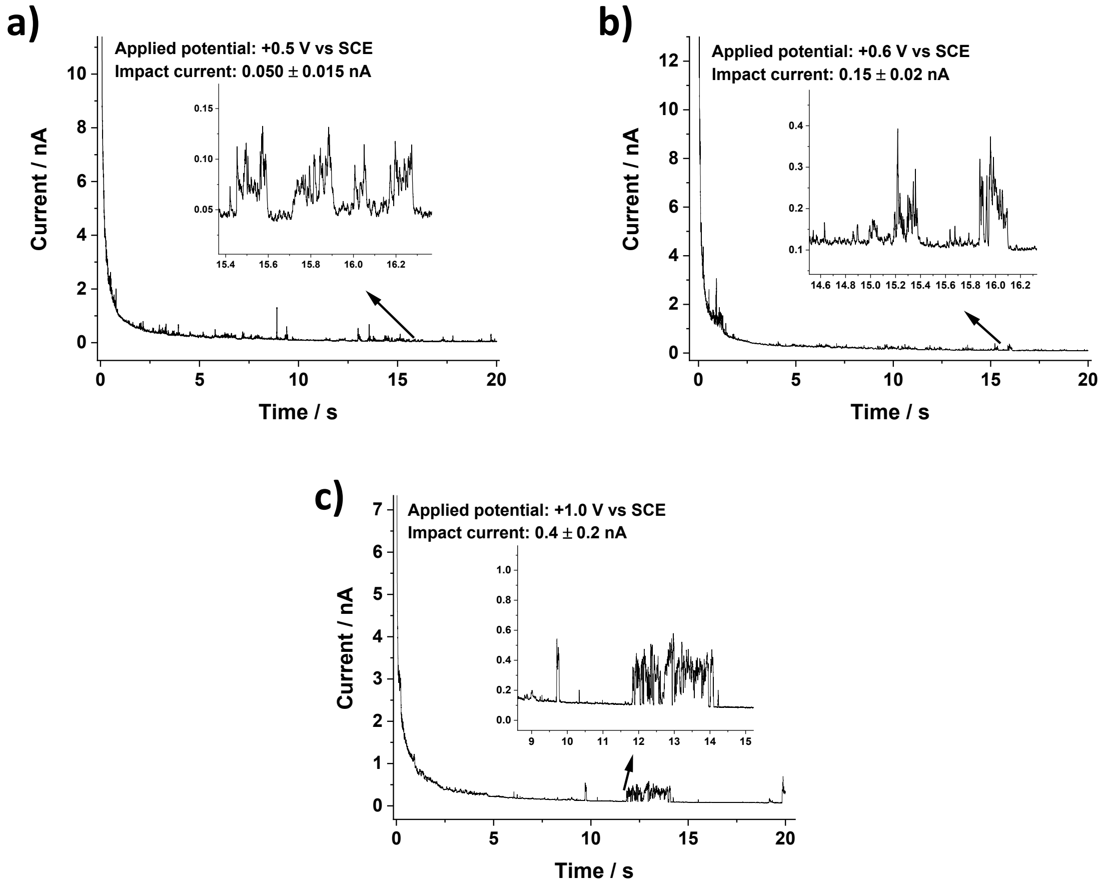

Figure S6. Chronoamperograms showing representative impact signals in 0.1 mM HR with 0.05 M BR solution at applied potentials of a) +0.5 V vs SCE, b) +0.6 V vs SCE and c) +1.0 V vs SCE.

Section 8. 'Nano-impacts' in the absence of 4-hexylresorcinol

In this section chronoamperograms in the absence of b-MWCNTs is given showing no impact signals.

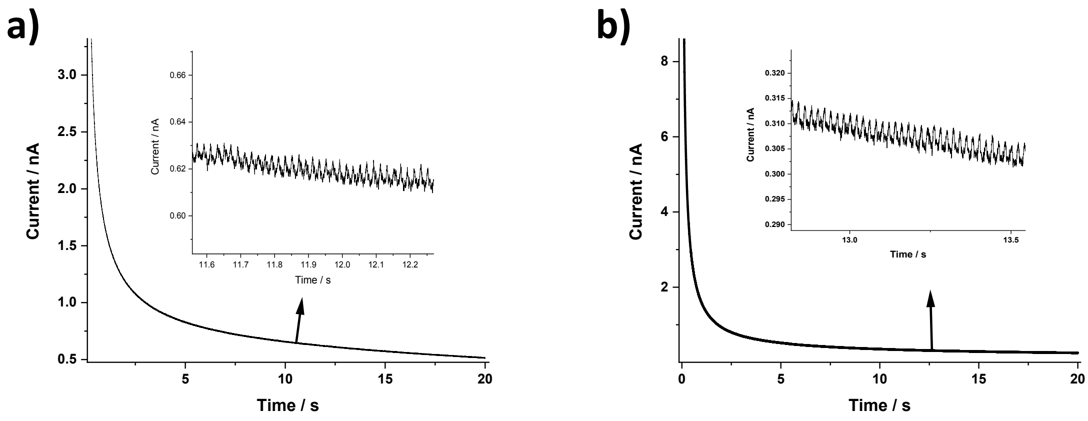

Figure S7. Chronoamperogram without b-MWCNTs at applied potential of +1.0 V vs SCE, a) only 0.05 M BR buffer solution and b) in 0.1 mM hexylresorcinol with 0.05 M BR buffer solution

Section 9. Chronoamperograms showing impact currents at various applied potential in 0.05 M BR buffer solution with b-MWCNTs

In this section chronoamperograms showing impact signals in 0.05 M BR buffer solution is given at different applied potentials.

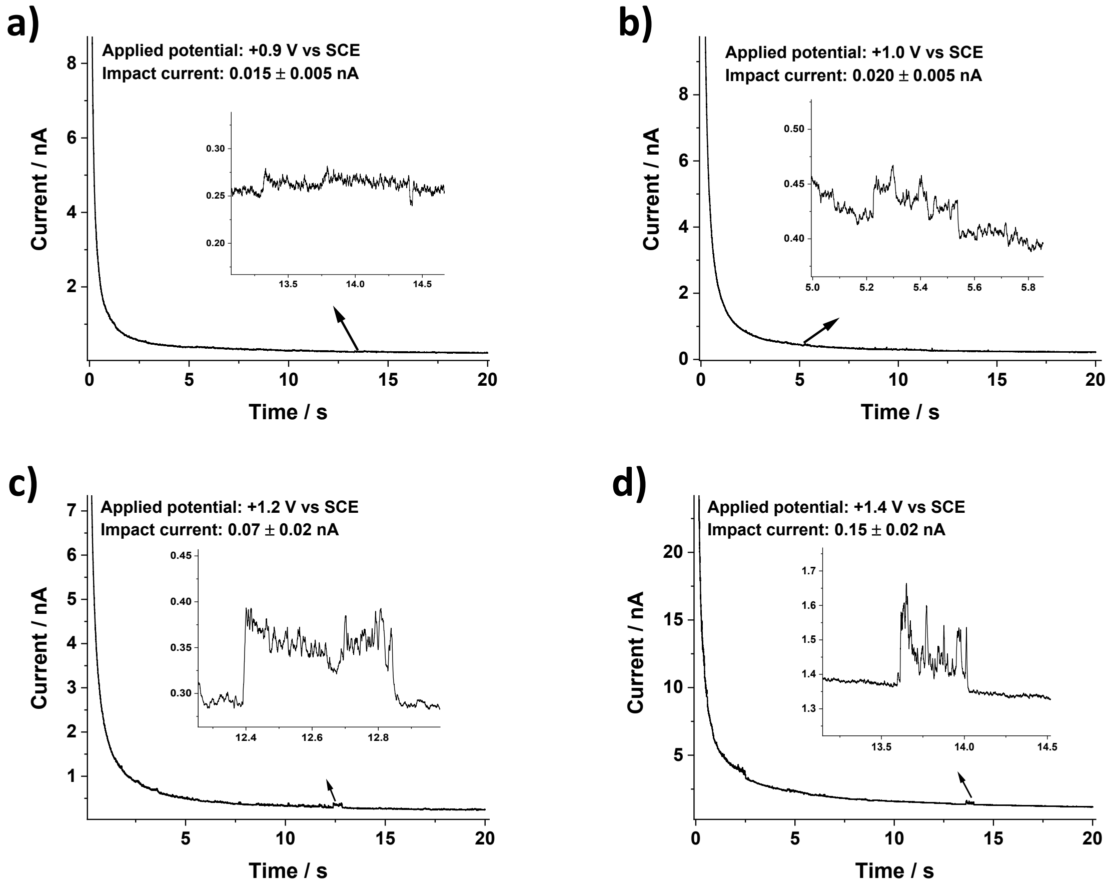

Figure S8. Chronoamperograms showing representative impact signals in only 0.05 M BR solution at applied potentials of a) +0.9 V vs SCE, b) +1.0 V vs SCE, c) +1.2 V vs SCE and d) +1.4 V vs SCE.

Section 10. Interpreting impact signals as adsorptive in origin

To analyse if the impact current obtained from the impacts of single b-MWCNTs were adsorptive or diffusional, first the assumption was made for the adsorption of HR onto the surface of single b-MWCNT. To estimate the number of monolayers of adsorption three differently possible orientations of adsorptions were considered, flat view, edge view and end view.

Then the maximum surface coverage is calculated using the area of the molecule. Note that the bond length, bond angle and van der Waals radii were obtained from ChemDraw 3D.

The maximum surface coverage, Γ_{max} is given by,

$$\Gamma_{max} = \frac{1}{N_A S_A}$$

where, N_A is the Avogadro constant and S_A is the surface area of the molecule.

The surface area of the molecule can be calculated by viewing the molecule in three different possible ways it can get adsorbed on the surface of the b-MWCNTs, namely flat view, edge view and end view.

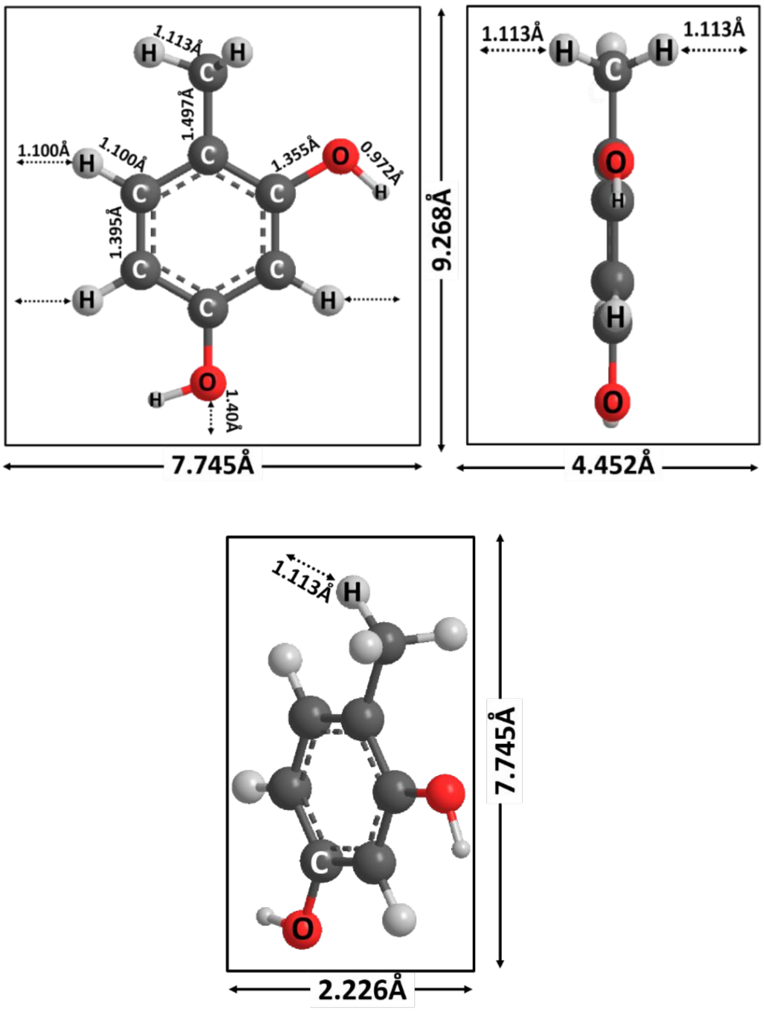

Figure S9: Rectangular box model of 4-hexylresorcinol showing flat view, edge view and end wise view.

Note that here the area of the molecule is calculated considering that the molecule to occupy rectangular area along its length and width.

Hence the area of the molecule with their corresponding view is given as,

1) Flat view (SFV): 7.18 (±0.04) x 10^{-15} cm^2
2) Edge view (SEdV): 4.13 (±0.05) x 10^{-15} cm^2
3) Endwise view (SEV): 1.45 (±0.11) x 10^{-15} cm^2

Thus, the Γ_{max} is found to be,

1) Flat view (Γ_{max} FV): 2.31 (±0.12) x 10^{-10} mol cm^2
2) Edge view (Γ_{max} EV): 4.02 (±0.13) x 10^{-10} mol cm^2
3) Endwise view (Γ_{max} EV): 1.19 (±0.23) x 10^{-9} mol cm^2

Hence, the charge contributed by each orientation for a monolayer adsorption at the surface of the b-MWCNTs can be calculated using the following equation,

$$\Gamma = \frac{Q}{nFA}$$

where, Γ is the surface coverage in mol cm^{-2}, Q is the charge calculated form the impact currents in C, n is the number of electrons, F is the Faraday constant in C mol^{-1} and A is the area of the single b-MWCNT.

Thus, the charge at each orientation is,

1) Flat view: 3.78 (±0.02) x 10^{-13} C
2) Edge view: 6.59 (±0.06) x 10^{-13} C
3) End view: 1.95 (±0.09) x 10^{-12} C

The average charge calculated from the impact current in 0.1 mM, 0.2 mM and 0.4 mM of HR is 7.54 (±1.50) x 10^{-12} C, 1.52 (±1.35) x 10^{-11} C and 3.05 (±1.44) x 10^{-11} C respectively.

Hence the number of monolayers of HR adsorbed on the b-MWCNTs for each orientation is given in Table S1, assuming a close packed arrangement. As discussed in the main text these are implausibly large so the origin of the signals in adsorptive behaviour is discounted.

Table S1. No. of monolayers of HR adsorbed at single b-MWCNTs at various concentrations assuming the currents are due to adsorbed HR (but see text for refutation of this assumption)

Concentration / mM	No. of monolayers		
	Flat view	Edge view	End view
0.1	20 (±2)	11 (±3)	4 (±3.5)
0.2	40 (±2)	23 (±3)	8 (±3.5)
0.4	81 (±2)	46 (±3)	16 (±3.5)

Section 11. Interpreting impacts as diffusional signals from 4-hexylresorcinol

As, explained in section 10, next the impact signals were considered to be diffusion-controlled mass-transport, hence the diffusion coefficient of HR was calculated and compared with expectations based on literature values (see Section 12).

For this calculation, the MWCNT is considered to resemble a cylindrical electrode [3] with length *l* and radius *r*, the current for time *t* is given by equation [1].

$$I_{ss} = 2\pi nFDClf(\tau)$$

with,

$$f(\tau) = \frac{e^{-\sqrt{\pi\tau}/20}}{\sqrt{\pi\tau}} + \frac{1}{ln\left[(2e^{-\gamma}\tau)^{1/2} + e^{-5/3}\right]}$$

$$\tau = \frac{4Dt}{r^2}$$

where the value of γ = 0.5772, *D* is the diffusion coefficient, *C* is the analyte concentration, *n* is the number of electrons (n = 2), *t* impact duration.

The slope of I_{ss} versus concentration is obtained from Figure 3b; inset (main text).

Hence the diffusion coefficient of 4-hexylresorcinol is calculated to be 4.04 (± 2.26) x 10^{-6} cm^2 s^{-1} by assuming that the impact signals are diffusive. Comparison, as discussed in the main text, with literature values given in the following section suggests that this is a plausible interpretation of the impact signal data.

Section 12. Literature on the diffusion coefficients of quinones in aqueous solutions

Table S2. Diffusion coefficients of different quinones in aqueous solutions at 298 (±3) K

Solution	Diffusion co-efficient/ $cm^2\ s^{-1}$	References
1 mM hydroquinone in 1 M sulfuric acid	5.0×10^{-6}	[4]
0.5 mM anthraquinone-2-sulfonate in 0.1 M KOH	5.7×10^{-6}	[5]
5 mM p-benzoquinone in 0.5 M KCl	7.0×10^{-6}	[6]
1 mM 2,5-dihydroxy-1,4-benzoquinone in 1 M KOH	3.7×10^{-6}	[7]
1 mM 9,10-anthraquinone-2,7-disulfonic acid in 1 M H2SO4	4.4×10^{-6}	[8]
1 mM anthaquinone-2-sulfonate in 0.1 M NaOH and 0.1 M KCl	4.7×10^{-6}	[9]
1 mM anthraquinone-2,6-disulfonate in 0.1 M NaOH and 0.1 M KCl	5.2×10^{-6}	[9]

Note that the literature values were rounded to two significant figures.

References

[1] J. Ellison, C. Batchelor-McAuley, K. Tschulik, R.G. Compton, The Use Of Cylindrical Micro-Wire Electrodes For Nano-Impact Experiments; Facilitating The Sub-Picomolar Detection Of Single Nanoparticles, Sensor Actuat B-Chem 200 (2014) 47-52.
[2] Nano-Lab Inc., Multiwalled carbon nanotubes, 2018.
[3] X.T. Li, C. Batchelor-McAuley, S.A.I. Whitby, K. Tschulik, L.D. Shao, R.G. Compton, Single Nanoparticle Voltammetry: Contact Modulation of the Mediated Current, Angew Chem Int Edit 55(13) (2016) 4296-4299.
[4] B. Yang, L. Hoober-Burkhardt, F. Wang, G.K.S. Prakash, S.R. Narayanan, An Inexpensive Aqueous Flow Battery for Large-Scale Electrical Energy Storage Based on Water-Soluble Organic Redox Couples, J Electrochem Soc 161(9) (2014) A1371-A1380.
[5] Q. Li, C. Batchelor-McAuley, N.S. Lawrence, R.S. Hartshorne, R.G. Compton, Electrolyte Tuning Of Electrode Potentials: The One Electron Vs. Two Electron Reduction Of Anthraquinone-2-Sulfonate In Aqueous Media, Chem Commun 47(41) (2011) 11426-11428.
[6] Y.H. Tang, Y.R. Wu, Z.H. Wang, Spectroelectrochemistry For Electroreduction Of P-Benzoquinone In Unbuffered Aqueous Solution, J Electrochem Soc 148(4) (2001) E133-E138.
[7] Z.J. Yang, L.C. Tong, D.P. Tabor, E.S. Beh, M.A. Goulet, D. De Porcellinis, A. Aspuru-Guzik, R.G. Gordon, M.J. Aziz, Alkaline Benzoquinone Aqueous Flow Battery for Large-Scale Storage of Electrical Energy, Adv Energy Mater 8(8) (2018).
[8] C. Wiberg, T.J. Carney, F. Brushett, E. Ahlberg, E.G. Wang, Dimerization of 9,10-anthraquinone-2,7-Disulfonic acid (AQDS), Electrochim Acta 317 (2019) 478-485.
[9] C. Batchelor-McAuley, Q. Li, S.M. Dapin, R.G. Compton, Voltammetric Characterization of DNA Intercalators across the Full pH Range: Anthraquinone-2,6-disulfonate and Anthraquinone-2-sulfonate, J Phys Chem B 114(11) (2010) 4094-4100.
